# Feasibility and Acceptability of a Co‐Designed Self‐Management Programme for People Living With Kidney Failure

**DOI:** 10.1111/jorc.70051

**Published:** 2026-02-16

**Authors:** Laura E. Lunardi, Richard K. Le Leu, Richard Bastin, Paul N. Bennett, Fiona Donnelly, Monique Borlace, Jie Zeng, David Myers, Merrilyn Bradbrook, David Bradbrook, Rhanee Lester, Effie Johns, Anne Britton, Shilpanjali Jesudason, Shyamsundar Muthuramalingam, Dorothea Dumuid, Lisa A. Matricciani

**Affiliations:** ^1^ Central Northern Adelaide Renal and Transplantation Service, Royal Adelaide Hospital Adelaide South Australia Australia; ^2^ College of Health Adelaide University Adelaide South Australia Australia; ^3^ School of Psychology, College of Education, Behavioural and Social Sciences Adelaide University Adelaide South Australia Australia; ^4^ CNARTS, CALHN Consumer Reference Group Adelaide South Australia Australia; ^5^ School of Nursing and Midwifery Griffith University Brisbane Queensland Australia; ^6^ Alliance for Research in Exercise Nutrition and Activity (ARENA), College of Health Adelaide University Adelaide South Australia Australia

**Keywords:** feasibility, kidney failure, patient activation, self‐management

## Abstract

**Background:**

Effective self‐management is critical in chronic kidney disease, yet many patients report low confidence and engagement in healthcare. Co‐designed self‐management programmes may strengthen uptake and sustainability, but few have been tested in kidney failure not yet on dialysis populations. This study evaluated the feasibility and acceptability of a self‐management programme designed by and for people with chronic kidney disease.

**Methods:**

We conducted a single‐arm, pre–post feasibility study in adults with kidney failure (eGFR ≤ 15 mL/min/1.73 m²) not yet on dialysis at an Australian nephrology unit. Patients attending the clinic were invited to participate in a 12‐week nurse‐led self‐management programme, co‐designed in partnership with people with kidney disease. The programme included motivational interviewing, structured goal setting, and tailored education delivered via digital or paper format. Feasibility measures included participant recruitment and retention, programme adherence and acceptability, assessed through a post‐programme evaluation survey.

**Results:**

Of the 40 patients invited, 31 consented to enrol on the programme (recruitment rate 31/40, 78%). All but one participant completed the follow‐up survey (30/31, retention rate 97%), achieving 100% programme adherence among completers. Most participants reported improvements in knowledge, greater confidence in self‐management, and valued the nurse support. Participants highlighted the convenience of home‐based learning but noted challenges with small‐screen readability and requested additional video resources.

**Conclusion:**

A co‐designed, nurse‐led self‐management programme was feasible and highly acceptable for people with kidney failure not yet on dialysis. Findings support further evaluation in a larger controlled trial. Integration of this programme into routine care could strengthen patient readiness for kidney replacement therapy.

## 1 Introduction

Chronic kidney disease (CKD) is a progressive condition associated with substantial symptom burden, reduced quality of life, increased morbidity and higher healthcare costs (Deng et al. 2025; Evans et al. 2022; Kidney Health Australia 2020a; Stenvinkel et al. 2020). Effective self‐management is essential to delay progression, manage complications, and prepare for kidney replacement therapy in the event of kidney failure (Chen et al. 2011; Magadi et al. 2022).

A key determinant of effective self‐management is patient activation, defined as an individual's knowledge, skills and confidence to manage their own health (Hibbard et al. 2004). Patients with higher activation levels are more likely to engage in self‐care, have fewer unmet medical care needs, and have better communication with health professionals (Hibbard et al. 2004). In contrast, people with lower activation levels are more likely to be hospitalised and have poorer adherence to treatment and greater health costs (Chen et al. 2011; Novak et al. 2013). Despite this, a recent survey of patients with kidney failure not receiving dialysis found that most patients (73%) reported low activation levels (Lunardi et al. 2024), highlighting a vulnerable population with substantial unmet self‐management needs and reinforcing the importance of developing interventions that explicitly target patient activation in this setting.

Interventions designed to strengthen patient activation and self‐management skills have the potential to improve preparedness for kidney replacement therapy and enhance patient‐centred care (Hussein et al. 2021; Lightfoot et al. 2022). Increasingly, co‐design approaches, which actively involve people with lived experience as partners in research, are recognised as a means of improving relevance and sustainability of healthcare interventions, including self‐management interventions for chronic diseases (Cazzolli et al. 2024). However, the effectiveness of such interventions depends not only on their content, but also on their relevance, acceptability and feasibility within real‐world clinical settings (Kelly et al. 2020). While patient activation interventions in people with chronic diseases have been shown to be acceptable and feasible across 26 studies involving 2610 patients (Lin et al. 2020), it remains unknown how patients with CKD perceive such interventions.

Accordingly, this study aims to evaluate the feasibility and acceptability of a co‐designed, nurse‐led, patient‐activation‐guided self‐management programme for people living with kidney failure not yet on dialysis. By embedding consumers as partners throughout intervention development, this study sought to address known limitations of previous CKD self‐management programmes and provide foundational evidence to support future large‐scale trials.

## 2 Literature Review

Self‐management interventions in CKD have been widely studied, with evidence suggesting that structured education, goal setting, action planning, and problem‐solving can improve knowledge, self‐efficacy, and selected behavioural outcomes (Lunardi et al. 2023). Patient activation has emerged as a particularly important construct, with higher activation levels consistently associated with better self‐management behaviours and reduced healthcare utilisation across the CKD population (Hibbard et al. 2004; Hussein et al. 2022; Lunardi et al. 2024). However, the effectiveness of CKD self‐management interventions has been variable, and many programmes have reported challenges with implementation and sustained engagement (Donald et al. 2018; Reston et al. 2023; Welch et al. 2015; Williams et al. 2012). Importantly, relatively few studies have focused on people with kidney failure not yet receiving dialysis, despite this group facing heightened uncertainty, increasing symptom burden, and complex decision‐making demands.

Although numerous CKD self‐management programmes have been developed, many have struggled with feasibility and acceptability in practice (Reston et al. 2023; Williams et al. 2012). Reported barriers include high participant burden, limited accommodation for varying health literacy levels, digital usability challenges, cultural inappropriateness, and insufficient integration into routine clinical care. These limitations have contributed to variable uptake, high attrition rates, and reduced real‐world impact.

A critical limitation of the existing literature is that most interventions have been designed using traditional, clinician‐led approaches, with patients involved primarily as study participants rather than as partners in intervention development (Donald et al. 2018; Scholes‐Robertson et al. 2024; Tong et al. 2008). This may be why programmes fail to adequately reflect patients' lived experiences, priorities, and contextual constraints, and limit their relevance and acceptability.

Co‐design is an approach that actively involves patients, caregivers, clinicians, and researchers as equal partners throughout the research process (Cazzolli et al. 2024; Tong et al. 2008). By valuing lived experience as a form of expertise, co‐design aims to ensure that interventions are responsive to real‐world needs, culturally appropriate, and accessible across diverse populations.

A recent review of 19 randomised controlled trials of patient activation interventions in CKD found that the most successful self‐management programmes included tailored patient education, goal setting with an action plan, and problem‐solving exercises (Lunardi et al. 2023). However, none of these trials adopted an authentic co‐designed approach that actively involved people with a living experience of kidney disease in the development of the intervention. This is a critical gap because interventions developed without meaningful consumer input often fail to address the real‐world needs, barriers and priorities of people living with CKD (Donald et al. 2018).

Evidence from the Better Evidence and Translation in CKD (BEAT‐CKD) programme highlights the importance of involving patients and caregivers as equal partners in research, not only as participants (Cazzolli et al. 2024). A co‐design approach ensures that programmes reflect diverse lived experiences, cultural context and health literacy needs, which can strengthen engagement, equity, sustainability and long‐term implementation.

Evidence from other chronic conditions further supports the value of co‐design (Sumner et al. 2024). In diabetes, heart failure, and chronic obstructive pulmonary disease, co‐designed or culturally tailored self‐management programmes have demonstrated higher retention, stronger acceptability, and improved patient‐reported outcomes compared with standard approaches (Federman et al. 2024; Santos et al. 2023; Yehualashet et al. 2024). Key features of successful co‐designed interventions include flexible delivery formats, peer support, personalised goal setting, and integration within routine care, elements consistently prioritised by patients but often underemphasised in traditional programme designs (Bombard et al. 2018; Slattery et al. 2020).

Despite growing recognition of the benefits of co‐design, a clear gap remains in the CKD literature (Donald et al. 2018). To our knowledge, no published studies have co‐developed and tested self‐management programmes tailoring patient activation for people with kidney failure not yet receiving dialysis using a co‐design methodology.

This study addresses this gap by co‐developing and testing a nurse‐led self‐management programme for feasibility and acceptability. By focusing on a population with high unmet needs and low activation levels, this research provides important preliminary evidence to inform future effectiveness trials and the broader implementation of co‐designed self‐management interventions in kidney care.

## 3 Materials and Methods

### 3.1 Study Design and Participants

We conducted a single‐arm, pre‐post feasibility study at the Central Northern Adelaide Renal and Transplantation Service (CNARTS), the largest renal service in South Australia that delivers care across multiple clinic locations. The study was conducted from March to August 2025 and followed the modified CONSORT extension for single‐arm pilot and feasibility trials (Supporting Information: S2) (Eldridge et al. 2016). Eligible participants were adults (≥ 18 years) with kidney failure (estimated glomerular filtration rate [eGFR] ≤ 15 mL/min/1.73 m²) not yet receiving dialysis. Exclusion criteria included the inability to communicate in English or a cognitive impairment that impeded the ability to provide informed consent. The CKD clinic at CNARTS is composed of nurses and nurse practitioners. They work collaboratively with nephrologists and allied health clinicians to provide a single point of contact for patients with advanced CKD (stages 4–5) to support their decision‐making about the different kidney treatment options. The CKD clinic at the time of this study consisted of 119 patients with kidney failure not yet receiving dialysis. A consecutive sampling method was used to invite the first 40 participants attending the CKD clinic on 30th March who met the eligibility criteria to take part in this study, following an in‐person appointment with their nurse practitioner.

### 3.2 Ethics Statement

Before data collection commenced, potential participants were approached in person during clinic attendance, by phone, or via email by the study investigator or clinical staff. Participants who expressed interest were then referred to the study investigator, who obtained informed consent and enroled participants in the trial. Patients were advised that participation was voluntary and did not affect clinical care. Participants were explicitly advised of their right to withdraw from the study at any time without any negative consequences. The study was conducted in accordance with the National Statement on the Ethical Conduct of Human Research (2023).

### 3.3 Intervention

**Figure 1:**
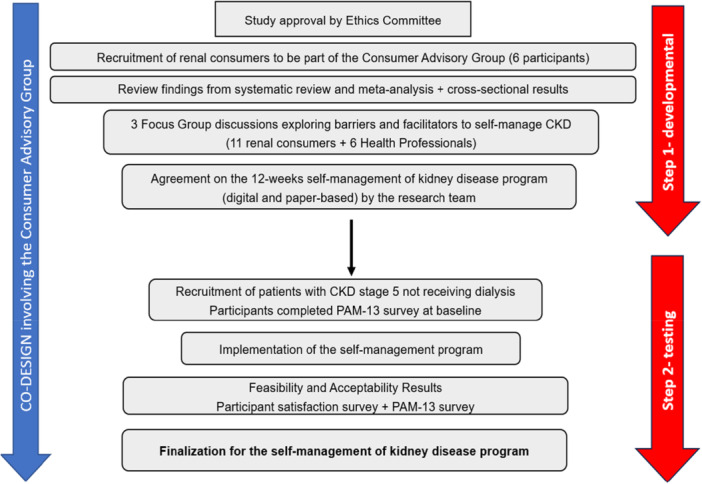
Co‐design process of the self‐management programme – Flow chart diagram.

The intervention was developed through a co‐design process, led by patients living with CKD in partnership with nephrology clinicians and researchers. This process aimed to ensure consumer priorities and lived experiences informed the content, delivery and format of the programme, while also providing strategic direction for the project. As presented in Figure 1, a Consumer Advisory Group (CAG) was developed following ethics approval. The CAG consisted of six people living with CKD, recruited from the Central Adelaide Local Health Network (CALHN) Consumer Partnering and Community Engagement in South Australia, who participated as co‐investigators. The CAG deemed it important to understand the enablers and facilitators of self‐management from both patient and clinician perspectives, leading to a qualitative focus group study involving 11 renal consumers and 6 nephrology clinicians (Lunardi et al. 2025). Members of the CAG assisted in the development of the interview guide and facilitated the focus group interviews. A central underpinning of these discussions was that consumers valued self‐management programmes that were tailored to the emotional, informational, and practical needs of each patient, with flexible learning formats (digital and paper‐based), and that included opportunities for peer support (Lunardi et al. 2025).

Drawing on the findings of the qualitative focus group study, as well as a comprehensive review (Lunardi et al. 2023) and cross‐sectional study (Lunardi et al. 2024) and existing programmes (Donald et al. 2021; Kelly et al. 2020; Lightfoot et al. 2024; Young et al. 2024), the CAG guided the development of the self‐management programme. Through review of these resources and discussions, the CAG guided the content, structure and delivery of the programme. This included decisions around its length of 12 weeks, the availability of education modules in digital and print formats and offering face‐to‐face sessions with a nephrology nurse practitioner (Weeks 1 and 12), phone coaching (Weeks 3 and 6), and a peer group session at Week 9 (Supporting Information: S1). Consumers also reviewed the online platform through which the programme would be accessed and contributed to the development of resources including educational materials and videos from Kidney Health Australia (Kidney Health Australia 2020b). The four core elements included in the programme were: (1) *motivational interviewing* (MI) to enhance readiness and confidence; (2) *structured goal setting*, identifying personalised health goals; and (3) *educational modules* covering kidney disease, treatment options, lifestyle and self‐care (Supporting Information: S1), and (4) *peer support*, including consumer‐led group discussions to share experiences and practical strategies for health living.

This intervention relied on MI as this approach can provide a patient‐centred approach that supports behaviour change, enhances self‐efficacy, and facilitates shared decision‐making, which are critical in preparing for kidney replacement therapy (Ok and Kutlu 2021; Sanders et al. 2013). The nurse practitioners received a 1‐day‐3‐h in‐person and online MI training prior to the commencement of this study by a renal psychologist with formal MI training. Training included practical skills‐based components.

### 3.4 Variables and Data Collection

Demographic characteristics collected at baseline included: age (years), sex (female vs. male), current living situation (with family vs. alone) and living location (metropolitan Adelaide vs. regional), as per the 2021 Australian Bureau of Statistics (Australian Bureau of Statistics 2021).

#### 3.4.1 Primary Outcomes

The primary outcomes of this trial were feasibility and acceptability. Feasibility was assessed in terms of: (1) *eligibility rate*, defined as the number of participants meeting eligibility criteria out of the total number of people attending the clinics; (2) *recruitment rate*, defined as proportion of invited participants accepting to participate; (3) *retention*, defined as the number of enroled participants completing the 12‐week programme; (4) *adherence*, defined as the number of enroled participants that completed all modules and surveys.

Programme acceptability was assessed using Likert‐scale questionnaires. Items were rated on either a 0–10 scale (with endpoints labelled) or a 1–5‐point scale (with all points labelled). Where applicable, scales were reverse‐coded so that extreme values could be consistently interpreted, with lower values reflecting greater programme acceptability. Favourable responses were defined a priori based on the direction of each scale. For 0–10 items, lower numeric scores (≤ 3) were classified as favourable perceptions (e.g., higher perceived value, lower burden, more likely to recommend the programme). For 5‐point Likert items, favourable responses were defined as (≤ 2), where ‘agree or strongly agree’ or ‘good/very good’ were selected. Responses are presented and summarised as frequencies and percentages, with medians reported as measures of central tendency.

Programme acceptability was assessed using eight statements. Participants were asked three questions related to study value, burden and enjoyment using the following 11 point Likert scale questions: ‘On a scale of 0 to 10, please rate the value of this study (0 = extremely valuable and 10 = not valuable’; ‘On a scale of 0 to 10, please rate the burden of this study (0 = not very burdensome/required little effort and 10 = highly burdensome/required a lot of effort)’; and, ‘On the scale of 0 to 10, how likely would you recommend this program to others? (0 = very likely and 10 = very unlikely)’. This style of question was pragmatically selected as it has been used in other feasibility studies (Murphy et al. 2024). However, it lacks formal validation, which is recognised as a minor limitation of this study. A further five statements used 5‐point Likert scale (each rated from 1 = strongly agree to 5 = strongly disagree) were related to participants' perceptions of the programme and its impact on their self‐management of their CKD following this programme: Programme Support: ‘The level of support from your renal nurse coordinator was adequate’; Information: ‘The information was provided at a level that I found easy to understand’; Knowledge: ‘The program has helped me to better understand my kidney condition’; Confidence: ‘I feel more confident in managing my kidney condition’; Group Session: ‘I found the in‐person group session to be worthwhile’; and, Overall Experience using digital platform: ‘Rate your overall experience using this digital program in terms of ease of use’. Lastly, two open‐ended questions were used to explore programme strengths and areas for improvements *What did you like best about the self‐management program?* and *What did you like least about the program?*

#### 3.4.2 Secondary Outcome

The secondary outcome measure was the change in patient activation, determined using the 13‐item Patient Activation Measure (PAM‐13) (Insignia Health 2017), which has been validated among patients with CKD (Lightfoot et al. 2021). PAM scores range from 0 to 100, with higher values reflecting higher activation. Patient activation classifications were determined using established cut‐off points (Insignia Health 2017). Level 1 (0.0–47.0) represents low activation, suggesting that the person does not yet understand their role in healthcare; Level 2 (47.1–55.1) indicates that the person does not yet have the knowledge and confidence to take action; Level 3 (55.2–67) indicates that the person is beginning to engage in positive health behaviours; Level 4 (67.1–100) indicates that the person is proactive and engaged in recommended health behaviours. (Hibbard et al. 2004; Hussein et al. 2021; Insignia Health 2017). The PAM‐13 was administered at baseline and upon completion of the self‐management programme. Change in patient activation was calculated as the difference between pre‐ and post‐ intervention PAM score, with positive values reflecting an increase in patient activation.

### 3.5 Data Analysis

Feasibility measures were reported using descriptive statistics as appropriate. A priori feasibility criteria included > 50% recruitment, > 80% retention and > 50% adherence, consistent with prior similar studies (Golestaneh et al. 2023; Kyte et al. 2022). Acceptability measures were reported using descriptive statistics and presented in a graphical format to aid interpretation. The number (and percentage) of participants within each PAM‐13 level was reported. Descriptive summary statistics (mean and standard deviation [SD]) were used to report baseline, follow‐up and mean changes in PAM‐13 scores for each of the four levels of activation. We computed 95% confidence intervals (CIs) for mean changes and calculated effect sizes using Cohen's *d* with values of 0.2, 0.5 and 0.8 representing small, medium and large effects, respectively (Gignac and Szodorai 2016). Data were analysed using the statistical software SPSS Version 25.0 (IBM Armonk, NY: 2017).

## 4 Results

**Figure 2:**
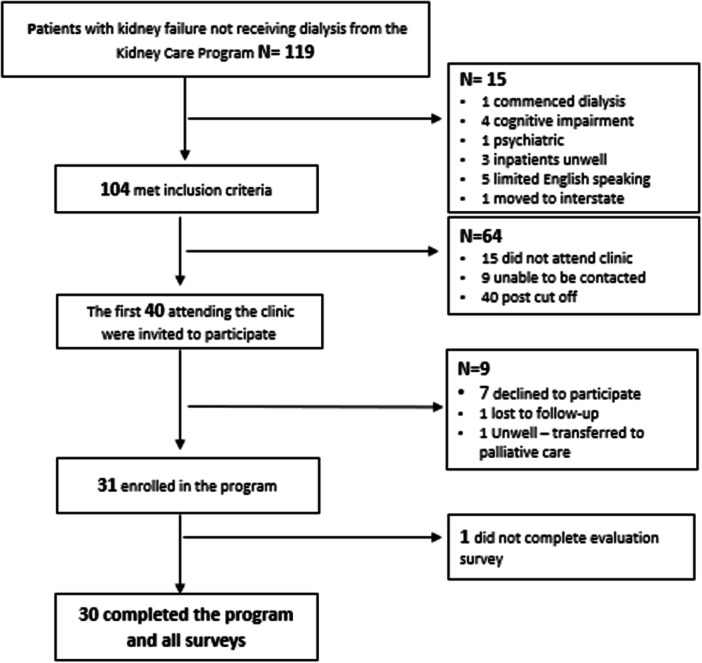
Modified CONSORT flow diagram.

Of 119 patients screened, 104 met the eligibility criteria (eligibility rate 87%), and the first 40 consecutive kidney care clinic attendees were enroled. Recruitment ceased at 40, as this represented the pre‐specified sample size for the feasibility trial. Patients attending the kidney care clinic were invited to participate (Figure 2), and 31 participants provided written informed consent and enroled in the trial (recruitment rate 78%).

### 4.1 Participant Characteristics

Participants' median age was 73 years (range: 45–80), and 36% were female. Most participants lived with family (83%) and resided in metropolitan areas (74%) (Table 1). Baseline characteristics are reported for participants who completed both baseline and follow‐up assessments (*n* = 30).

**Table 1:** Baseline characteristics of participants (*N* = 30).

Variables of renal consumers (*N* = 30)	*n* (%)
Age (year)	
Median (range)	73 (45–80 years)
Female	12 (36.4)
Current living situation	
With family	25 (83)
Alone	5 (17)
Location	
Metropolitan	22 (73.5)
Regional	8 (26.5)
PAM‐13 (mean/SD)	67.5 (±16.1)

### 4.2 Study Feasibility

As shown in Table 2, all feasibility measures met a priori criteria. Most participants invited to participate provided consent to participate (recruitment rate: 31/40, 78%). One participant completed all modules but did not complete the programme evaluation survey (retention rate: 97%), resulting in 30 participants who completed the 12‐week intervention. Programme adherence was high, with all 30 participants completing all six modules and the satisfaction surveys within 12 weeks.

**Table 2:** Feasibility outcomes.

Domain	Prespecified progression criterion	Observed rate
Eligibility	≥ 60%	87%
Recruitment	≥ 50%	78%
Retention	≥ 80%	97%
Adherence	≥ 50%	100%

Most participants (*n* = 22, 71%) accessed the programme via the digital platform, while 29% accessed the paper‐based materials. Of those who completed the digital platform, the average completion time was 10 weeks, with all users reporting that their experience using the online programme was very good (*n* = 20) or good (*n* = 2).

### 4.3 Study Acceptability

Overall, the programme's acceptability was rated positively by participants (Table 3 and Supporting Information: S4). Of all participants, 90% reported the programme to be of high value, and 97% reported that participation was a low burden. Similarly, 97% indicated they were likely to recommend the programme to others.

**Table 3:** Acceptability of the intervention (Likert‐scale responses).

Item	Scale	*n*	Mean	Median (IQR)	Favourable responses, *n* (%)
Perceived value of the study	0–10 (lower = more valuable)	30	1.63	1 (1–2)	27 (90%)
Perceived burden of the study	0–10 (lower = less burdensome)	30	1.57	1 (1–2)	28 (93%)
Likelihood of recommending programme	0–10 (lower = more likely to recommend)	30	1.27	1 (1–2)	29 (97%)
Support from renal nurse coordinator was adequate	1–5 (lower = strongly agree)	30	1.27	1 (1–2)	30 (100%)
Information was easy to understand	1–5 (lower = strongly agree)	30	1.33	1 (1–2)	30 (100%)
Programme improved understanding of kidney condition	1–5 (lower = strongly agree)	30	1.33	1 (1–2)	29 (97%)
Confidence in managing kidney condition	1–5 (lower = strongly agree)	30	1.5	1.5 (1–2)	30 (100%)
In‐person group session worthwhile	1–5 (lower = strongly agree)	8	1.5	1.5 (1–2)	8 (100%)
Ease of use of digital programme	1–5 (lower = very good)	22	1.09	1 (1–1)	22 (100%)

**Figure 3:**
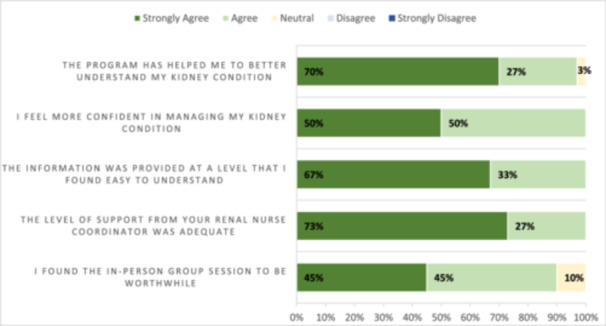
Programme acceptability ratings.

As shown in Figure 3, nearly all participants agreed or strongly agreed that the programme improved their knowledge (97%) and found the group session very useful (90%). All participants agreed and strongly agreed that the programme increased their confidence in self‐managing their CKD; they highly valued the nurse practitioner support, which was considered to provide an adequate level of support.

Five participants provided feedback regarding what they enjoyed most about the programme; four provided comments related to how they enjoyed learning about their conditions (e.g., ‘I learned more about understanding my CKD effects and how to manage it better’), while one liked that they could undertake the programme at home. Only one participant provided a comment regarding what they liked least about the programme; this participant suggested, ‘It can be hard to read the information on a phone screen and a laptop is better for easy reading’. Two participants suggested improvements: adding more information about kidney treatment options and providing a magnification tool for phone users.

### 4.4 Patient Activation

Among 30 participants with paired PAM‐13 levels (1–4) and scores (1–100), the mean baseline score was 67.5 (SD: ± 16.1), and the mean follow‐up score was 66.1 (SD: ± 13.6). The mean change was −1.4 (SD: 17.8), 95% CI of −8.0 to 5.2, corresponding to a small effect size (Cohen's *d* = −0.08; *p* = 0.66). There was no missing data in the sample of participants who completed both surveys.

Table 4 shows baseline and follow‐up activation levels for each activation level. Of participants at levels 1–4 at baseline, only 50%, 33%, 77% and 55% remained at their respective levels at follow‐up. Change from baseline to a higher level of activation at follow‐up was noted in 50%, 67% and 15% of participants at levels 1–3 at baseline, respectively. Change from baseline to a lower level of activation was noted in 0% (participants with level 2 at baseline), 8% (participants with level 3 at baseline) and 44% (participants with level 4 at baseline). Overall, 7 participants (23%) improved by at least one level, 18 (60%) remained stable (suggesting maintenance of engagement), and 5 (17%) decreased by one activation level. Although prior work has proposed a minimal clinically important difference (MCID) for the PAM‐13 in some clinical populations (Wilkinson et al. 2025), no MCID has been formally validated in a population with CKD. Consequently, changes in patient activation levels in this study should be interpreted as exploratory rather than definitive evidence of clinical benefit.

**Table 4:** Patient activation outcomes.

Changes in PAM‐13 scores across baseline activation levels^a^						
Baseline activation levels	*N* (%)	Baseline score (mean ± SD)	Follow‐up score (mean ± SD)	Change in score (mean ± SD)	95% CI for change	Effect size (Cohen's *d*)
Level 1	2 (7)	44.6 ± 3.4	50.4 ± 7.9	5.8 ± 4.9	−38.2 to 49.8	1.18
Level 2	6 (20)	51.4 ± 1.6	63.6 ± 16.6	12.2 ± 12.4	−0.8 to 25.2	0.98
Level 3	13 (43)	64.8 ± 5.7	66.9 ± 11.6	2.1 ± 8.8	−3.2 to 7.4	0.24
Level 4	9 (30)	87.2 ± 10.9	69.8 ± 14.8	−17.4 ± 14.9	−28.9 to −5.9	−1.17

Changes in activation levels from baseline^b^					
Baseline activation levels		Follow‐up activation levels, *N* (%)			
	*N* (%)	Level 1	Level 2	Level 3	Level 4
Level 1	2	1 (50)	0	1	0
Level 2	6	0	2 (33)	2	2
Level 3	13	0	1	10 (77)	2
Level 4	9	1	1	2	5 (55)

^a^ Values are presented as mean ± SD.

^b^ Values are presented as numbers (%).

## 5 Discussion

This study demonstrated that our co‐designed self‐management programme for adults with kidney failure not yet on dialysis is both feasible and acceptable. Recruitment and retention rates exceeded prespecified feasibility measures, with 94% of enroled participants completing the programme and 100% adherence among completers. Participants reported improved knowledge, greater confidence in managing their kidney condition, and valued the convenience of flexible, home‐based delivery combined with ongoing nurse practitioner support. Digital delivery was feasible, with two‐thirds of participants preferring digital resources. This underscores the importance of maintaining flexible, hybrid delivery models to ensure equity, supporting accessibility across the metropolitan and regional CKD population. Consumer Advisors' suggestions, such as adding videos, improving digital readability, and offering optional one‐to‐one support, highlight opportunities to further enhance programme engagement and impact, which in turn may improve activation scores in future iterations. These findings highlight the potential of embedding co‐designed, nurse‐led, hybrid delivery approach programmes into routine renal care may improve patient preparedness for kidney replacement therapies and support informed decision‐making.

Importantly, while mean PAM‐13 scores did not significantly improve, nearly a quarter of participants increased by at least one activation level, suggesting an exploratory finding rather than a clinical benefit in a subset of patients. However, there is no literature‐based MCID for PAM‐13 in the CKD pre‐dialysis population. Interestingly, nearly half of the participants with the highest baseline activation (level 4) declined to a lower level at follow‐up. Several explanations are possible. Regression to the mean may account for some of this shift, as participants with very high scores at baseline had less scope for improvement. Second, sustaining motivation among already highly activated patients may be more challenging if programme content feels less targeted to their advanced knowledge and skills. Finally, the increased disease burden and psychosocial stressors in people with kidney failure approaching dialysis initiation may reduce engagement for some patients despite initial confidence. Similar declines have been reported in other feasibility studies in the haemodialysis population, where high baseline PAM scores proved difficult to maintain over time (Hussein et al. 2022). This finding reinforces the need for adaptive programme components that can sustain motivation across the activation spectrum.

Feasibility studies are critical because they identify what works in practice before full implementation. Self‐management programmes for CKD populations are not always feasible; this can depend on design, delivery methods, patient populations, and healthcare settings (Reston et al. 2023; Williams et al. 2012). Previous CKD self‐management programmes have demonstrated variable feasibility and engagement outcomes. A Multilingual, Culturally and Linguistically Diverse Populations self‐management programme (Williams et al. 2012) and a Multichannel Digital and Telephone Support self‐management programme for newly diagnosed CKD patients (Reston et al. 2023) were found to not be feasible due to high attrition rates related to language barriers, cultural nuances and literacy limitations, poor illness awareness (unaware they have CKD), technical complexities and cumbersome questionnaires (over 100 items), all of which severely limited uptake and retention. Both studies reported challenges with recruitment and sustained participation, with dropout rates exceeding 20% despite strong programme content. By contrast, several CKD self‐management interventions integrated into routine care, with flexible delivery, have demonstrated feasibility. The Kidney BEAM programme (a digital physical activity and wellbeing platform) demonstrated high recruitment across multiple sites and strong participant engagement, with participants valuing the blend of live and on‐demand sessions (Young et al. 2024). Similarly, the My Kidneys My Health web‐based tool was found to be highly acceptable, with users reporting high ease of use, usefulness, and an intention to continue engaging with the resource (Donald et al. 2021). The telehealth‐delivered coaching programme conducted in Australia also demonstrated feasibility, with good retention (93%) (Kelly et al. 2019) and improved self‐management outcomes in a real‐world renal clinic setting (Kelly et al. 2020).

In comparison with previous programmes, our study achieved excellent feasibility and acceptability outcomes, with 94% retention and 100% adherence among participants from both metropolitan and regional areas. This achievement suggests that the co‐design process and integration of consumer‐led peer support may have been critical enablers of engagement, including cultural tailoring and delivery models focused on patients' needs and priorities. While mean activation scores did not significantly increase, the finding that nearly a quarter of participants improved by at least one level suggests clinical benefit for a substantial proportion of the sample, particularly among those with lower baseline activation levels. This may reflect the added value of consumer‐led facilitation and the integration of patient‐identified priorities into the programme design.

Beyond nephrology, recent feasibility self‐management trials in diabetes (Yehualashet et al. 2024), heart failure (Santos et al. 2023) and COPD (Federman et al. 2024) have shown strong retention and high acceptability, especially when programmes are co‐designed or culturally tailored, include peer support, and integrate practical components (e.g., coaching) within routine care/community settings. Echoing our finding that most of the participants choosing digital delivery one‐third of participants preferred paper‐based materials, our self‐management of kidney disease programme highlighted the importance of tailored delivery modes and flexible formats to maximise accessibility required for patients to take a more active role in self‐manage their CKD. Taken together with these non‐renal examples, our study demonstrates that a co‐designed and consumer‐led approach in advanced CKD is both practical and effective in routine care and may explain the unusually high engagement levels relative to previous kidney‐specific interventions. Larger controlled trials are now needed to determine effectiveness in improving self‐management, sustaining activation, and reducing unplanned dialysis starts.

### 5.1 Strengths and Limitations

Strengths of this study include an authentic co‐designed self‐management programme involving consumers as partners in programme development, a hybrid delivery method that enables people from regional areas to access the programme, and integration into routine kidney care. Several limitations should be acknowledged. This study was conducted with a small and homogeneous sample, limiting generalisability. The lack of a control group, the short follow‐up and reliance on self‐reported acceptability measures further constrain the interpretation of the findings. Also, the 10‐point Likert scale used to assess acceptability has not undergone formal reliability/validation. This study population had minimal representation from culturally and linguistically diverse (CALD) groups; all participants were English‐speaking, predominantly highly educated and health‐literate people living in metropolitan areas. This self‐selection bias limits the applicability of the findings to a more diverse and underserved population, including individuals with low health literacy or from CALD backgrounds. Furthermore, the MI training provided to nurse practitioners by the renal psychologist was not a certified course and fidelity scoring was not conducted.

Accordingly, the finding should be considered preliminary. A larger, multi‐site controlled trial is required to establish effectiveness and improve generalisability. In addition, this study was not powered to detect changes in PAM‐13 scores; therefore, effectiveness outcomes should be interpreted with caution.

## 6 Implication for Clinical Practice

Embedding co‐designed, nurse‐led programmes that combine MI, structured goal setting, flexible education delivery (digital or paper‐based) and consumer‐led peer support within standard care pathways may help prepare patients for kidney replacement therapy and improve knowledge and confidence to optimise self‐management. The use of consumer‐led peer support may enhance engagement, particularly for patients with lower activation levels.

Importantly, participant feedback identified digital usability barriers, including difficulty reading content on small screens. To maximise clinical impact, services should consider adopting hybrid delivery models that accommodate both digital and non‐digital users, incorporating universal design principles (Australian Human Rights Commission 2025). This includes the use of responsive digital design, adjustable text size and magnification options, and the continued availability of paper‐based resources. Incorporating ongoing patient feedback to optimise programme content, such as video resources and accessibility tools, and providing ongoing training for renal nurses in MI techniques are also recommended.

Wider implementation of this approach has the potential to improve patient preparedness for dialysis initiation, support shared decision‐making, and ultimately reduce unplanned dialysis starts and associated healthcare costs.

## 7 Conclusion

This study demonstrates that a co‐designed self‐management programme is feasible to deliver within routine kidney care and is highly acceptable to people with kidney failure not yet on dialysis. Embedding this programme as part of standard care could strengthen patient readiness for kidney replacement therapy. These findings support further evaluation of the programme in a larger controlled trial to determine its effectiveness in improving self‐management, patient activation, and preparedness for kidney replacement therapy.

## Author Contributions

Laura E. Lunardi, a PhD student, renal consultant, and nephrology nurse practitioner, conceived and designed the study in collaboration with Merrilyn Bradbrook, David Bradbrook, Richard Bastin, David Myers, Rhanee Lester, Effie Johns, and renal consumers with over 20 years of lived experience with CKD who provide support with conceptualisation and data curation. Fiona Donnelly, Monique Borlace, and Jie Zeng, nurse practitioners, contributed to data curation and programme delivery. Anne Britton, nurse lead, and Shilpanjali Jesudason, renal consultant and head of the renal department, contributed to data curation, resource development and software. Paul N. Bennett, Lisa A. Matricciani, Rhanee Lester, and Shyamsundar Muthuramalingam, senior researchers, contributed to supervision, funding acquisition, investigation, methodology, formal analysis and visualisation. Laura E. Lunardi drafted the initial manuscript, which was reviewed and edited by all co‐authors. All authors contributed to the study and approved the final manuscript.

## Ethics Statement

The study protocol was approved by the CALHN research committee (reference number 19031) and the institutional review board at the University of South Australia (Human Research Ethics Application ID 206246). This study was registered with the Australian New Zealand Clinical Trials Registry (ANZCRT) #12625000136404.

## Conflicts of Interest

The authors declare no conflicts of interest.

## Supporting information

Self‐management of kidney disease – Program delivery.

CONSORT extension Pilot and Feasibility Trials.

Patient Activation Measure ‐13 (PAM‐13).

Acceptability measures, n (%).

## Data Availability

The data supporting the findings of this study are available from the corresponding author upon reasonable request. The data are not publicly available due to privacy or ethical restrictions.
