# Supplementary material for: Feasibility and Acceptability of a Co‐Designed Self‐Management Programme for People Living With Kidney Failure
Source: J Ren Care. 2026 Feb 16;52(1):e70051. doi: 10.1111/jorc.70051 (PMC12908431; doi:10.1111/jorc.70051)
Supplement: Supplementary file 1 — Self‐management of kidney disease – Program delivery. [file JORC-52-0-s004.docx]

**Suplementary Material:** Self-manageme of kidney disease – Program Deliverly

| **Weeks** | **Type of Sessions** | **Delivery method** | **Agenda of coaching session / activities** | **Facilitator** | **Duration** |
| --- | --- | --- | --- | --- | --- |
| Week 1 | Session 1 | Individual activity   1. min) | ***Self-Management Program***   - Overview - Digital or booklet - Discuss meetings schedule. - Completion of PAM-13 survey | Nephrology Nurse Practitioner | 15’ |
|  |  |  | ***Interactive Education (booklet)***   - How kidneys work |  | 20’ |
|  |  |  | ***Goal setting and Action plan***   - Partnership concept. - What do you currently find challenging or difficult living with CKD? - What to do when you are sick - Setting up 2 individual goals and action plan for the next 2 weeks. |  | 25’ |
| Week  3 | Session 2 | Individual activity (phone)  (30 min) | - Introduction and recap of the previous session | Nephrology Nurse Practitioner | 5’ |
|  |  |  | ***Interactive Education (booklet)***   - Chronic kidney disease |  | 15’ |
|  |  |  | ***Goal setting and action plan:***   - Review of the 2 individual goals and action plan - Completion of IPOS-renal version |  | 10’ |
| Week 6 | Session 3 | Individual activity (patient preference (phone or TEAMs or in-person)  (30 min) | - Introduction and recap of the previous session | Nephrology Nurse Practitioner | 5’ |
|  |  |  | ***Interactive Education (booklet)***   - What can be done to improve kidney health. |  | 15’ |
|  |  |  | ***Goal setting and action plan:***   - Review of the 2 individual goals and action plan - Motivational interview about Becoming independent. |  | 10’ |
| Week  9 | Session 4 | Coaching / motivational discussion  Group or individual (patient preference)  (90 min) | - Introduction of the participants - Aim of this meeting: To learn self-management through sharing experience of living with chronic kidney disease. | Nurse Practitioner | 10’ |
|  |  |  | ***Interactive Education (booklet)***   - Practical ways to make healthy choices - Self-assessment of CKD symptoms. |  | 30’ |
|  |  |  | ***Dynamic problem-solving***   - Choosing a problem(s) /challenge(s) - Action plan / discuss capacity to cope with it. |  | 50’ |
| Week  12 | Session 5 | Individual activity  In-person  (60 min) | - Introduction and recap of the previous session | Nurse Practitioner | 5’ |
|  |  |  | ***Interactive Education (booklet)***   - Resume of previous sessions - Concept of Self-Advocacy |  | 25’ |
|  |  |  | ***Problem-solving, goal setting and action plan***   - Strengths and needs - Strategies for long-term plan |  | 20’ |
|  |  |  | **Completion of the Program**   - Your Kidney Team and Finding More Information - Discussion about the “Preparing for Dialysis Toolkit” (handed out in earlier appointments with the kidney care team): *Supporting you to get ready for Dialysis.* - Completion of PAM-13 and feedback survey |  | 10’ |

1. **Program delivery format**


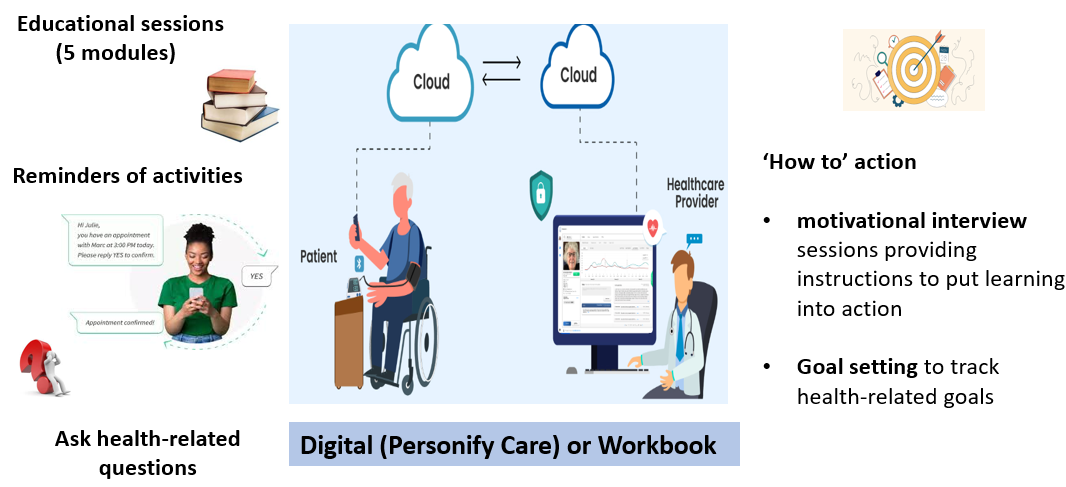


1. **12-weeks self-management program timeline**
