# Supplementary figures and images for: Feasibility and Acceptability of a Co‐Designed Self‐Management Programme for People Living With Kidney Failure
Source: J Ren Care. 2026 Feb 16;52(1):e70051. doi: 10.1111/jorc.70051 (PMC12908431; doi:10.1111/jorc.70051)

1. **Suplementatry Material**: Patient Activation Measure survey


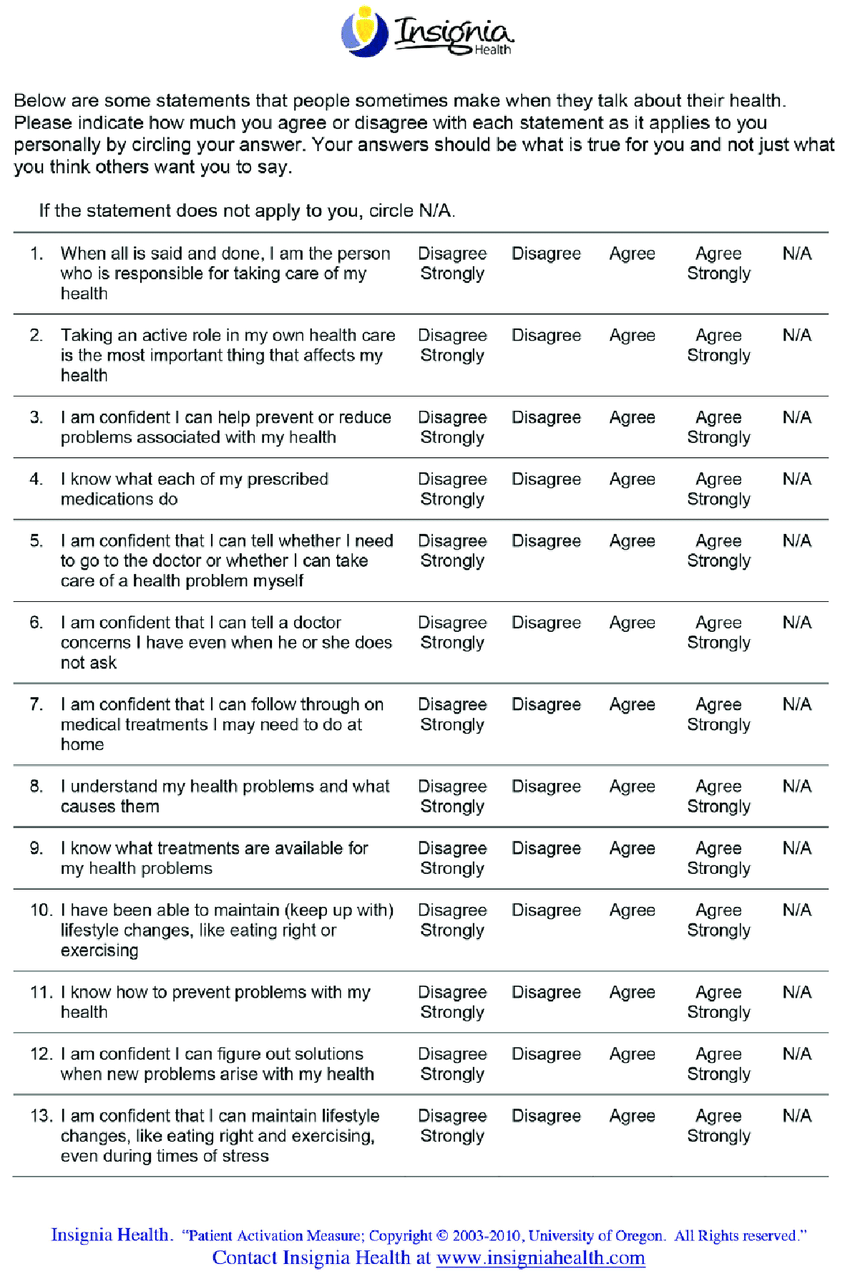

Supplement: Supplementary file 3 — Patient Activation Measure ‐13 (PAM‐13). [file JORC-52-0-s003.docx]
