# Supplementary material for: Feasibility and Acceptability of a Co‐Designed Self‐Management Programme for People Living With Kidney Failure
Source: J Ren Care. 2026 Feb 16;52(1):e70051. doi: 10.1111/jorc.70051 (PMC12908431; doi:10.1111/jorc.70051)
Supplement: Supplementary file 4 — Acceptability measures, n (%). [file JORC-52-0-s002.docx]

**Supplementary Material**: Acceptability measures, n (%)

|  | *On a scale of 0 to 10, please rate the value of this study* | | | | | | | | | | | | | | | | Mean | Median |
| --- | --- | --- | --- | --- | --- | --- | --- | --- | --- | --- | --- | --- | --- | --- | --- | --- | --- | --- |
| Extremely  valuable | 0 | 1 | 2 | | 3 | 4 | | 5 | 6 | | 7 | 8 | | 9 | 10 | Not valuable | 1.63 | 1 |
|  | 0 | 20(67) | 7(24) | | 0 | 0 | | 3(10) | 0 | | 0 | 0 | | 0 | 0 |  |  |  |
|  | *On a scale of 0 to 10, please rate the burden of this study* | | | | | | | | | | | | | | |  |  |  |
| Not very burdensome | 0 | 1 | 2 | | 3 | 4 | | 5 | 6 | | 7 | 8 | | 9 | 10 | Highly burdensome | 1.57 | 1 |
|  | 0 | 22(74) | 6(20) | | 1(3) | 0 | | 0 | 0 | | 0 | 0 | | 0 | 1(3) |  |  |  |
|  | *On the scale of 0 to 10, how likely would you recommend this program to others?* | | | | | | | | | | | | | | |  |  |  |
| Very likely | 0 | 1 | 2 | | 3 | 4 | | 5 | 6 | | 7 | 8 | | 9 | 10 | Very unlikely | 1.27 | 1 |
|  | 0 | 24(80) | 5(17) | | 0 | 1(3) | | 0 | 0 | | 0 | 0 | | 0 | 0 |  |  |  |
|  | *The level of support from your renal nurse coordinator was adequate* | | | | | | | | | | | | | | |  |  |  |
| Strongly agree | 1 | | | 2 | | | 3 | | | 4 | | | 5 | | | Strongly disagree | 1.27 | 1 |
|  | 22(73) | | | 8(27) | | | 0 | | | 0 | | | 0 | | |  |  |  |
|  | *The information was provided at a level that I found easy to understand* | | | | | | | | | | | | | | |  |  |  |
| Strongly agree | 1 | | | 2 | | | 3 | | | 4 | | | 5 | | | Strongly disagree | 1.33 | 1 |
|  | 20(67) | | | 10(33) | | | 0 | | | 0 | | | 0 | | |  |  |  |
|  | *The program has helped me to better understand my kidney condition* | | | | | | | | | | | | | | |  |  |  |
| Strongly agree | 1 | | | 2 | | | 3 | | | 4 | | | 5 | | | Strongly disagree | 1.33 | 1 |
|  | 21(70) | | | 8(27) | | | 1(3) | | | 0 | | | 0 | | |  |  |  |
|  | *I feel more confident in managing my kidney condition* | | | | | | | | | | | | | | |  |  |  |
| Strongly agree | 1 | | | 2 | | | 3 | | | 4 | | | 5 | | | Strongly disagree | 1.5 | 1.5 |
|  | 15(50) | | | 15(50) | | | 0 | | | 0 | | | 0 | | |  |  |  |
|  | *I found the in-person group session to be worthwhile.* ***n=8*** | | | | | | | | | | | | | | |  |  |  |
| Strongly agree | 1 | | | 2 | | | 3 | | | 4 | | | 5 | | | Strongly disagree | 1.5 | 1.5 |
|  | 4(44) | | | 4(44) | | | 0 | | | 0 | | | 0 | | |  |  |  |
|  | *Rate your overall experience using this digital program in terms of ease of use.* ***n=22*** | | | | | | | | | | | | | | |  |  |  |
| Very Good | 1 | | | 2 | | | 3 | | | 4 | | | 5 | | | Very poor | 1.09 | 1 |
|  | 20(91) | | | 2(9) | | | 0 | | | 0 | | | 0 | | |  |  |  |

**The first raw indicates the Likert value for each question and the second row indicate n (%)**
